# Two genetic loci control syllable sequences of ultrasonic courtship vocalizations in inbred mice

**DOI:** 10.1186/1471-2202-12-104

**Published:** 2011-10-21

**Authors:** Hansol Choi, Saegeun Park, Daesoo Kim

**Affiliations:** 1 Department of Biological Sciences, Korea Advanced Institute of Science and Technology (KAIST), Daejeon, 305-701, Korea

## Abstract

**Background:**

The ultrasonic vocalizations (USV) of courting male mice are known to possess a phonetic structure with a complex combination of several syllables. The genetic mechanisms underlying the syllable sequence organization were investigated.

**Results:**

This study compared syllable sequence organization in two inbred strains of mice, 129S4/SvJae (129) and C57BL6J (B6), and demonstrated that they possessed two mutually exclusive phenotypes. The 129S4/SvJae (129) strain frequently exhibited a "chevron-wave" USV pattern, which was characterized by the repetition of chevron-type syllables. The C57BL/6J strain produced a "staccato" USV pattern, which was characterized by the repetition of short-type syllables. An F1 strain obtained by crossing the 129S4/SvJae and C57BL/6J strains produced only the staccato phenotype. The chevron-wave and staccato phenotypes reappeared in the F2 generations, following the Mendelian law of independent assortment.

**Conclusions:**

These results suggest that two genetic loci control the organization of syllable sequences. These loci were occupied by the staccato and chevron-wave alleles in the B6 and 129 mouse strains, respectively. Recombination of these alleles might lead to the diversity of USV patterns produced by mice.

## Background

Male courtship sounds are an adapted trait for attracting females and increasing reproductive success [1]. Recently, mice were found to emit ultrasonic vocalizations (USVs) in response to females or their urine [2-5]. USVs were shown to be functional in attracting females in play-back experiments [6,7].

Mice are an important model for the genetics of behavior; so mouse USVs have been a focus in studies of genetic mechanisms underlying animal communication. Mice knocked out for the FoxP2, Drd2, or vasopressin receptor genes are known to produce abnormal USVs in terms of the length, frequency, and number of sound emissions [8-10], suggesting that genetic factors contribute to the development of vocalization pathways [10].

Beyond these quantitative traits, recent studies have focused on the qualitative mechanisms underlying the temporal and spectral features of mouse USVs. It was shown that the courtship USV of male mice has a song-like structure, which is composed of syllables in discrete categories where the syllables are arranged in a sequential order with a preferred transition between the syllables [11].

The syllable repertoire of bird song is modulated by environmental factors, but recent cross-fostering experiments indicate that mouse USVs are genetic [12]. However, it is not known how genetic mechanisms are involved in the organization of specific USV sequences.

Here, we compared the syllable patterns of courtship USVs between 129 and B6 mice, which are the most common genetic backgrounds used in gene-targeting experiments. We describe the existence of two genetic loci that follow the rules of Mendelian inheritance and control the selection of specific syllables, thereby contributing to the diversity of courtship USVs.

## Results

### Different syllable organizations in 129 and B6 strains of mice

Male mice USVs were induced by introducing a B6 female at estrus stage. Male mice exhibited sniffing, chasing, and mounting behaviors, as previously reported [13]. USVs emitted during sniffing behaviors were compared to avoid potentially confounding effects with different behaviors [14].

**Figure 1:**
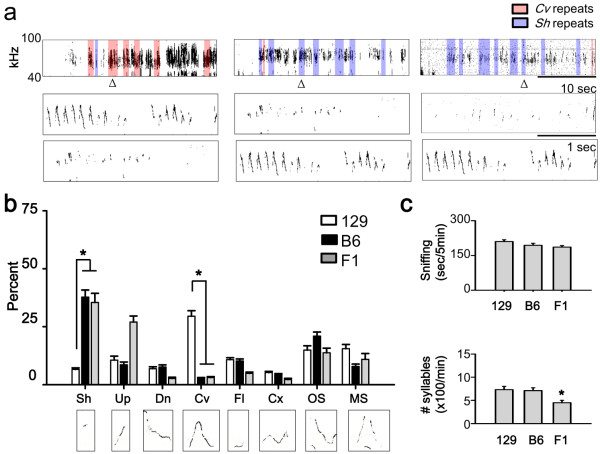
**Syllabic composition of the USVs of 129, B6, and F1 mice**. (a) *up*: staccato (short repeats) (red) and chevron-wave (chevron repeats) (blue) of 129 (*left*), B6 (*center*), and F1 (*right*). The box represents short or chevron adjacently repeated regions. *down*: detailed view of the area in the triangle. (b) The ratio of each syllable type. 129 and B6 exhibited different use of short and chevron. F1 had a similar syllable composition to B6, but differed from 129. *down*: representative sonogram for each syllable produced by 129. * *p *< 0.01. (c) Sniffing and total number of syllables during sniffing. * *p *< 0.05.

USV patterns were characterized by visual inspection of sonograms and it was found that129 and B6 males had mutually exclusive phenotypes. Based on previously published criteria for syllable classification [10,11,15,16] it was determined that 129 mice frequently repeated chevron-type syllables, whereas B6 mice used short-type syllables in their repeats (Figure 1a). The chevron-rich repeats are referred to as "chevron-wave" USVs, while the short-rich repeats are referred to as "staccato" USVs. These results suggest that B6 and 129 mice have different syllable preferences during the generation of courtship USVs.

Quantification of syllable composition indicated that chevron-type syllables were infrequently used by B6 mice, but they were dominant in 129 mice. Short-type syllables were significantly more commonly used by B6 mice compared with 129 mice (Figure 1b; *F*(14,476) = 30, *p *< 0.001 for strain × syllable type; for chevron or short, *p *< 0.001 for 129 *vs*. B6, Student-Newman-Keuls (SNK test) multiple comparison test), although they had the capacity to generate both syllable types (Figure 1b). Both strains showed no significant differences in the emission of other syllable types (Figure 1b and Table 1; *p *> 0.4 for *Sh*, *Up*, *Dn*, *Fl*, *Cx*, *OS*, or *MS*, for 129 *vs*. B6, SNK test).

**Table 1:** Parameters for syllables produced by 129, B6, and F1.

Average Peak Frequency				Duration			
**kHz**	**B6**	**F1**	**129**	**msec**	**B6**	**F1**	**129**

Sh*	75.2 ± 0.4	†72.2 ± 0.8‡	69.7 ± 1.0	Sh	7.4 ± 0.7	7.4 ± 0.7	8.5 ± 0.5
Up*	75.5 ± 0.4	†73.2 ± 0.5	70.7 ± 0.9	Up	23.4 ± 0.7	24.5 ± 1.0	30.1 ± 1.1
Dn*	73.0 ± 0.5	71.4 ± 1.2	68.9 ± 0.7	Dn	35.0 ± 0.2	38.5 ± 5.7	42.0 ± 1.6
Cv*	73.8 ± 0.5	76.3 ± 0.9	72.2 ± 0.7	Cv*	47.7 ± 3.0	57.4 ± 4.1	57.1 ± 1.4
Fl	73.2 ± 0.4	68.1 ± 0.9	65.1 ± 0.6	Fl	29.3 ± 1.1	27.4 ± 1.8	29.2 ± 8.7
Cx*	73.0 ± 0.5	70.6 ± 0.8	68.8 ± 0.7	Cx*	56.6 ± 3.1	65.1 ± 5.3‡	89.2 ± 3.2
OS*	71.9 ± 0.5	71.8 ± 1.1‡	64.6 ± 0.9	OS*	45.7 ± 4.5	45.1 ± 0.2‡	60.5 ± 1.9
MS*	70.0 ± 0.7	†63.1 ± 1.8	61.4 ± 0.9	MS	104.6 ± 9.4	†82.0 ± 2.4	95.8 ± 6.6

* p < 0.05 for B6 vs 129; † p < 0.05 for B6 vs F1; ‡ p < 0.05 for F1 vs 129

Average frequencies (*left*) and duration (*right*) of syllables compared among 129, B6, and F1. Overall, B6 had a higher average peak frequency than 129 males. F1 lay in the middle of the two. 129 produced longer Cv, Cx, and OS. F1 lay in the middle of the two.

Furthermore, there were no significant differences in the number of sniffs or syllables emitted, which reflects the motivational effect on USV emission (Figure 1c; sniffing: *p *= 0.52, *t*-test; number of syllables: *p *= 0.18, Student *t*-test). These results suggest that the chevron-wave and staccato phenotypes of B6 and 129 mice strains were associated with specific preferences in these strains for selecting chevrons or short syllables, despite their similar responsiveness in females.

### F1 males produce a B6-like USV

The genetic relationship between the staccato and chevron-wave phenotypes was addressed by generating F1 mice using both 129 ♀ × B6 ♂, and B6 ♀ × 129 ♂, matings. Interestingly, the proportion of short and chevron USV syllables produced by F1 males was similar to that produced by B6 males and differ from 129 (*F*(14,476) = 30.4, *p *< 0.001; short: *p *< 0.001 for 129 *vs*. B6 or F1 and *p *= 0.33 for B6 *vs*. F1; chevron: *p *< 0.001 for 129 *vs*. B6 and *p *= 0.86 for B6 *vs*. F1). The B6-like USVs of F1 mice were also unaffected by the paternal genotype (*p *= 0.47, B6 ♀ × 129 ♂, *vs*. 129 ♀ × B6 ♂,). A high proportion of frequently repeated short-type syllables was produced by the F1 mice (Figure 1b), as found in B6 mice (Figure 1b). F1 mice exhibited a similar amount of sniffing behavior (Figure 1c; *F*(2,60) = 2.5, *p *= 0.08) and emitted a lower number of syllables during sniffing behavior when compared with their parental strains (Figure 1c; ANOVA on ranks, *p *= 0.29; *p *< 0.05 for F1 *vs*. B6 or 129). These results suggest that staccato is the dominant phenotype, while chevron-wave is recessive.

### USV composition was not dependent on the female strain

**Figure 2:**
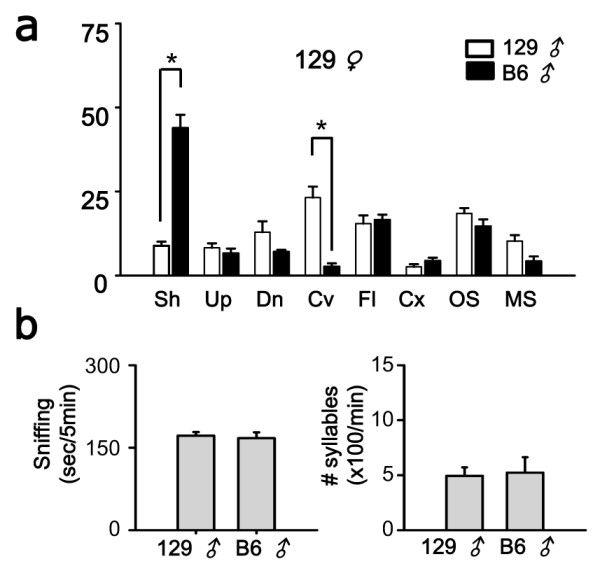
**Syllable composition of B6 and 129 males was not affected by the female strain**. (a) The ratio of each syllable type for 129 and B6 male mice stimulated by a 129 female. The syllable composition pattern was consistent with Fig. 1 (stimulated by B6 female). (b) Time spent sniffing (*left*) and total number of syllables (*right*) during sniffing behavior. B6 and 129 males stimulated by 129 female. * *p *< 0.01.

The different USV patterns of B6 and 129 males could be attributable to differences in their responsiveness to B6 females, *i.e*., B6 males might be exposed to females with the same genetic background, whereas 129 males might encounter females of different strains. To address this issue, we compared their responsiveness to 129 females. However, the relative syllable composition of each strain was not significantly affected by 129 females (Figure 2a; 129 male: *F*(1,210) = 0, *p *= 1, two way repeated measures ANOVA; *p *= 0.972 for 129 *vs*. B6 female, SNK test; B6 male: *F*(1,224) = 0, *p *= 1; *p *= 0.4 for 129 *vs*. B6, SNK test). These results suggest it was unlikely that differences in the USV phenotypes of B6 and 129 males (Figure 1) were due to motivational preferences associated with female stains.

### Repetition of a specific syllable in B6, 129 and F1mice

**Figure 3:**
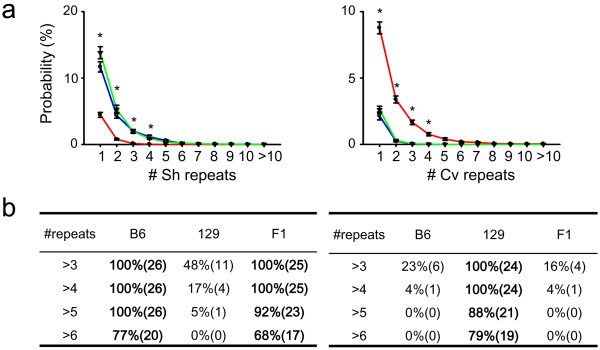
**Sequential syllable structure for USVs of B6, 129, and F1 mice**. (a) Syllable repetition in B6, 129, and F1 mice. The number of events of differing length was normalized with the total number of syllables. B6 and 129 mice emitted different amounts of short repetitions and chevron repetitions. B6-like phenotypes were dominant. * *p *< 0.01. (b) Proportions of animals with syllable repeats containing at three to six syllables.

To compare the degree of repetition of either short and chevron-type syllable among genotypes, we first measure the onset probability of those syllables along USV sequences (Figure 3a and 3b). Strain B6 or F1 produced more short repeats than 129 (Figure 3a*left*; *F*(2,710) = 165.1, *p *< 0.001; *p *< 0.001 for 129 *vs*. B6 or F1, *p *= 0.5 B6 *vs*. F1, SNK test; 1 ×-4 × short repeats, *p *< 0.001 for 129 *vs*. B6 or F1, SNK test). Chevron repeats were more frequent in 129 compared with B6 or F1 (Figure 3a*right*; *F*(2,710) = 50.0, *p *< 0.001; *p <*0.001 for 129 *vs*. B6 or F1, *p *= 0.14 for B6 *vs*. F1; 1 ×-4 × short repeats, *p *< 0.001 for 129 *vs*. B6 and F1, SNK method].

The biased preference for syllables was also found to be expressed in repeated syllables. For example, all B6 or F1 mice had the capacity to generate more than 4 × short repeats, whereas only 17% of the 129 mice produced 4 × short repeats (Figure 3b*left*; *p *< 0.05 for 129 *vs*. B6 or F1, chi-squared test). In contrast, all 129 males had the capacity to produce four or more chevron repeats (≥ 4 × chevron repeats), while few of the B6 (4%) or F1(4%) mice produced 4 × chevron repeats (Figure 3b*right*; *p *< 0.01 for 129 *vs*. B6 or F1, chi-squared test). These results suggest that the different USV patterns found in 129 and B6 mice (Figure 1 and 2) were associated with different syllable preferences of mice, both in terms of random selection and syllable repetition.

### Short-rich and chevron-rich phenotypes were segregated in F2 generations

**Figure 4:**
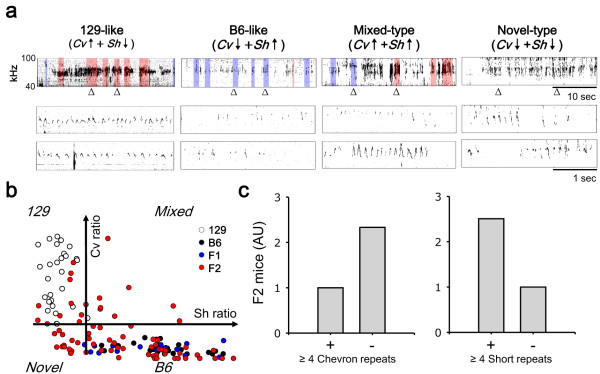
**F2 USVs were diversified into B6-like, 129-like, and mixed phenotypes**. (a) USV patterns of F2 mice. Representative sonograms of the four types of F2 mice are shown. Chevron repeat (red) and short repeats (blue) are highlighted (box). (b) Proportions of short (*x*-axis) and chevron (*y*-axis) for each mouse are shown. One dot is one mouse. Short and chevron compositions for B6 (blue) and F1 (green) overlapped, whereas 129 USVs (red) were discrete. F2 (black) mice were *B6-like *and *129-like*, containing both chevron and short rich repeats, while others lacked chevrons and short syllables. (c) The ratio of F2 mice with syllable repeats greater than four. Chevron-rich USVs were recessive, whereas short-rich USVs were dominant.

USVs of F2 mice obtained from the mating of F1 mice were analyzed to determine whether the mutually exclusive staccato and chevron-wave USV phenotypes were different alleles at the same genetic locus (Figure 4). B6 and F1 USVs were exclusively composed of short-type syllables, whereas the chevron-type was preferred by 129 mice (Figure 4b, white, black and red dots); however, F2 mice produced a more diverse composition of short-type and chevron-type syllables (Figure 4b, red dots). Many F2 mice emitted 129-like USVs (Figure 4a and 4b, 129-type), although a larger proportion of F2 mice produced B6-like USVs (B6-type). Furthermore, new types of mice were found that produced mixed-type USVs, while others lacked both staccato and chevron-wave repeats (novel-type) in their USVs. This suggests that at least two separate genetic loci determine the preference for syllable usage, *i.e*., one for the chevron-wave and another for the staccato repeat phenotypes.

Syllable repetition phenotypes were diversified also. Only c. 25% of the F2 mice exhibited the repetition of chevron syllables more than four times (Figure 4c; 12 of 41 total animals; χ^2 ^= 0.2, *p *= 0.65, chi-squared test, 1/4 *vs*. 12/41), so the allele for chevron repetition preference appears to be recessive. Approximately 75% of the F2 mice produced USVs containing short-rich syllables repeated more than four times (Figure 4c; 32 of 41; χ^2 ^= 0.07, *p *= 0.79 for chi-squared test 3/4 *vs*. 32/41), which indicates the dominance of this phenotype as also observed in the F1 generation.

## Discussion

Our results suggest that at least two genetic loci are responsible for the different syllable patterns of courtship USVs produced by 129 and B6 mice. Analysis of USVs in F1 mice showed that the chevron-rich USV repetition is a recessive phenotype, while the short-rich repetition is a dominant phenotype (Figure 1). The appearance of various combinations of those two phenotypes in the F2 generation suggests that these phenotypes are generated by two independent genetic loci (Figure 4). The two genetic loci are critical for the organization of syllable sequences in USVs. B6 and 129 mice share a similar ancestor [17], but allelic variations of genes and homozygosity for these alleles during the breeding of the two inbred strains may have led to the current phenotypes, which are defined by the redundant repetition of specific syllables.

What is the biological role of the two loci? It is unlikely that these loci control the capacity for the generation of specific syllables *per se *during prenatal development. If this hypothesis was true, adult B6 mice that are dominated by the usage of short-type syllables would be physically unable to generate chevron-type syllables. Previous reports show that B6 pups have no defects in the generation of specific types of syllables [18]. The current study also confirmed that adult B6 males can generate other types of syllables (Figure 1). Gene knockout studies also showed that the syllable repertoire mechanism can be dissociated from structural problems, *i.e.*, muscarinic receptor [10] or vasopressin 1b receptor [8] knockouts resulted in abnormal pitch, amplitude, or emission events when compared with wild-type mice, but there were no reported defects in terms of USV syllable structure.

A second hypothesis could explain the function of the two loci based on a paternal effect theory, which is used to explain the diversity of song patterns in songbirds. It is interesting to note that the syllabic makeup of emitted USVs changed as the mice aged. The USVs of B6 pups contained a larger proportion of chevron-type syllables (~40%) [16] compared with juvenile mice (2~3%) [15], as found in the current study (~1%). Furthermore, 129 mice exhibited an increase to ~30% chevron usage as adults, from only 10% chevron syllable usage as pups [18]. The age-dependent differentiation of the USV repertoire was not likely to be attributable to paternal effects, because the USV pattern of F1 mice was unaffected by the paternal genetic background (Figure 1 and 2).

A third hypothesis proposes that two loci are involved in the selection of syllables in the generation of USVs through an unknown mechanism. The results of the current study suggest that genetic recombination may be responsible for the composition of the USV syllable repertoire. Identifying the genetic component might help to understand the development of vocal communication in mice. It is plausible to assume that if selection is directed by multiple genes, then the combination of specific alleles may lead to complex patterns of USVs. This is consistent with the current study's observation of a diverse pattern of USVs in F2 mice, resulting from a B6 × 129 cross. B6-like and 129-like USV patterns were observed, but novel USV patterns with both or none alleles emerged in the F2 generation. This might be due to independent inheritance of the two alleles and reciprocal masking of recessive and dominant alleles by genetic recombination. The results of the current study suggest that genetic recombination might be responsible for the complexity of the USV repertoire, which is known to play a critical role in female choice and reproductive success. It remains to be elucidated whether USVs with repetition of the same type syllable are more efficient for attracting females when compared with USVs containing a complex combination of many syllables.

The F1 hybrid of B6 and 129 is suggested as an ideal genetic background for the study of gene knockout mice, because the effects of homozygous recessive mutations accumulate in the two inbred mice strains [19]. However, the current study suggests that F1 mice should be used carefully in USV analysis, because dominant mutations in B6 mice can produce a short-rich USV phenotype in the F1 genetic background (Figure 1). The biased USV phenotype of F1 mice due to the dominant mutation produces difficulties when evaluating the effectst of targeted gene on the expression of various syllables. To avoid this problem, various genetic backgrounds should be used in the analysis of mouse USVs with targeted mutations, as previously suggested [20].

The repetition of specific syllables is also seen in human speech disorders. Some patients with Tourette syndrome [21] or Parkinson's disease [22] exhibit palilalia, which is the continuous repetition of a phrase or specific syllables. Mechanisms underlying chevron-repeats or short-repeats might be shared by human speech disorders. Finally, the characterization of genes underlying syllable selection might provide profound insights into the evolutionary history of sound communication and the mechanism of human speech disorders.

## Conclusions

This study suggests that the selection of specific syllables in courtship USVs is controlled by a genetic mechanism in mice. At least two genetic loci are associated with the staccato and chevron-wave alleles of B6 and 129 mice, which control syllable preference combinations for these alleles and produce a diverse USV repertoire, even in the F2 generation. The diversification of the courtship USV repertoire in these alleles may contribute to the fitness of mice in nature. Further playback experiments to females with staccato and chevron-wave songs could help to explain this issue. Furthermore, the identification of these two loci will provide insights into the neural pathways that control the organization of syllable sequences and possibly aid the understanding of language disorders in humans.

## Methods

### Animal care and breeding

All procedures were performed according to the Korean Advanced Institute of Science and Technology (KAIST) guidelines for the care and use of laboratory animals and approved by the Institutional Animal Care and Use Committee (Protocol No. KA2008-28). Mice were maintained on a 12:12 h light:dark cycle (light cycle beginning at 06:00) at a temperature of 23°C. Food and water were accessible *ad libitum*. Male C57BL/6J (B6) and 129S4/SvJae (129) mice and their offspring were bred at KAIST (Daejeon, Korea). F1 mice were obtained from two types of breeding pairs: male B6 mice mated to 129 females and male 129 mice mated to B6 females. The data were collapsed across 129B6 (B6 was paternal) and B6129 (129 was paternal), because statistical analyses showed they were not significantly different. F2 mice were obtained from three breeding pairs composed of six F1 129B6 females and three F1 129B6 males. All male mice were sexually naïve and they had not mated before the test.

### Induction of courtship USVs

To induce USVs in male mice, a female was introduced into the male resident cage, because direct contact is known to be more effective in inducing USVs than female urine or indirect contact [10]. USV recordings were made with10~15-week-old male mice as follows: B6 to B6 female, n = 31; 129 to B6 female, n = 28; B6 to 129 female, n = 10; 129 to 129 female n = 9; F1, n = 30; and F2, n = 84. Each male was isolated in a transparent acrylic housing cage (140 × 210 × 130 mm) for at least three days before the recording of their courtship USVs. During the isolation period, male mice were exposed to the recording chamber for 30 min once every day. On the recording day, mice were placed in the recording chamber for 30 min. After a 5 min background recording, one randomly chosen estrus B6 (n = 30) or 129 (n = 10) female (3-5 months old, 4-5 mice housed together) was placed in the recording chamber [10]. The estrus stage was confirmed in female mice by visual inspection of the vagina [23]. A male was allowed to investigate the female for 5 min and emitted USVs were recorded. We used only males exhibiting high responsiveness to the females, as measured by the frequency of USVs (at least 100 events) and the frequency of sniffing behaviors (at least 150 sec, Figure 1).

### Recording of USV and courtship behavior

USVs were recorded through a ¼ inch microphone and amplified with a preamplifier and a main amplifier (sound recording from Brüel and Kjaer Inc., Denmark). Signals were filtered from 1 Hz to 100 kHz [24] and digitized with a sampling frequency of 250 kHz, 16 bits per sample, using a 1000 Hz high-pass digital filter (model 1322A, Axon Instruments, Union City, CA). Behaviors were simultaneously recorded with a CCD camera. Only 26/70 B6 or 129 mice exhibited mounting behavior during the 5 min recording time as small as average eight seconds. As infrequent to analysis, USVs recorded during non-sniffing behaviors were excluded from further analysis.

### Analysis of USV structure

Sound recordings were processed using a custom Matlab program. Short-time Fourier transform analysis was performed to draw sonograms (1,024 samples/block, 1/4 overlap, resulting in a time resolution of 1.02 ms and a frequency resolution of 0.45 kHz). Frequencies lower than 35 kHz were filtered out to reduce background white noise and audible squeaking from females. As a basal power spectrum, consecutive powers of 10 s without USVs were sampled and averaged for time, such that 1.2 times the basal power spectrum was subtracted from each power spectrum and frequencies with a power less than zero were set to zero. This procedure was used to reduce the background white noise [25].

USVs that containing more than 100 syllables were analyzed (B6 to B6 female, n = 26; 129 to 129 female, n = 24; B6 to 129 female, n = 9; 129 to 129 female, n = 8; F1, n = 25; F2, n = 70). Syllables were first identified based on their energy levels and then manually refined. For each time bin of the power spectrum, powers from 35 kHz to 100 kHz were summed and used as the USV power. At least two time bins (2.04 ms) with a USV power greater than the threshold were considered a syllable. The threshold was set manually to minimize false positive and false negative effects. Two syllables closer than 20 time bins (20.8 ms) were considered as one syllable.

The syllable classification method was similar to Grimsley *et al. *[26]. For the analysis of sound sequences, eight specific syllable types were classified by the dominant frequency change and the length, as follows: *short (Sh)*, shorter than 10 ms; *up frequency modulation (Up)*, increment of basal frequency *by *at least 10 kHz from beginning to end without a decrease larger than 5 kHz.;*down frequency modulation (Dn)*, decrease of basal frequency by at least 10 kHz from beginning to end without an increase larger than 5 kHz; *chevron (Cv)*, increase in pitch by at least 10 kHz followed by a decrease of at least 10 kHz without a subsequent increase larger than 6 kHz; *flat*, highest and lowest dominant frequency differed less than 10 kHz; *complex (Cx)*, continuous syllable without frequency steps not including the types above; *one step (OS)*, contained one frequency step (> 10 kHz basal frequency changes in 2.04 ms); and *multistep (MS)*, contained two or more frequency steps.

The degree of syllable repetition was defined here as the consecutive emission of the same syllable type without interruption by another type of syllable, or by a greater than 0.5 sec latency. The number of syllable repetition events was counted for each mouse and normalized by the total number of syllables.

### Statistical analysis

ANOVA with repeated measures was performed to analyze the strain-dependent differences in vocalization data of B6, 129, and F1 mice, with strain as a factor and call categories as repeated measures. This analysis was followed by the Student-Newman-Keuls test [16]. A chi-squared test was used to test the modes of inheritance in F2 mice.

## Authors' contributions

HC carried out the behavioral experiments, built the data analysis program, analyzed sound and behavior data, and drafted the manuscript. SP carried out the behavioral experiments, analyzed the sound and video data, and helped to draft the manuscript. DK conceived the study, participated in its design and coordination, and helped to draft the manuscript. All authors read and approved the final manuscript.
